# The Impact of Sunlight and Artificial Light at Night on Sleep Stages: Evidence From a 7-Day Sensor-Based Observational Study

**DOI:** 10.2196/75898

**Published:** 2026-06-05

**Authors:** Andrea Montanari, Li Min Wang, Amit Birenboim, Basile Chaix

**Affiliations:** 1 Sorbonne Université, INSERM, Institut Pierre Louis d’Epidémiologie et de Santé Publique IPLESP Paris France; 2 Department of Geography The Hebrew University of Jerusalem, Mt Scopus Jerusalem Israel; 3 The Center for Urban Innovation The Hebrew University of Jerusalem, Mt Scopus Jerusalem Israel

**Keywords:** circadian rhythm, electroencephalogram, light exposure, sleep, smartphone usage, wearable sensors

## Abstract

**Background:**

Exposure to circadian entrainers, such as sunlight, positively impacts sleep architecture, while exposure before bedtime to circadian disruptors, such as artificial light and smartphone use, can negatively affect sleep. However, real-world evidence from longitudinal observational studies that simultaneously capture these factors alongside electroencephalography-derived sleep stages remains limited.

**Objective:**

This study aimed to investigate the effects of specific environmental and behavioral factors on sleep metrics and architecture by using sensor-based measurements over 7 consecutive days. Specifically, it examined day-to-day associations between (1) daytime sunlight exposure and (2) prebedtime artificial light exposure and smartphone use with selected sleep outcomes on the following night.

**Methods:**

A total of 21 participants from the Jerusalem metropolitan area were monitored continuously using the Dreem wearable electroencephalography for sleep staging, HOBO data loggers for light exposure, the wGT3X+ triaxial accelerometer for physical activity, and a dedicated mobile app to record smartphone usage. Sleep outcomes included total sleep time (TST), sleep onset latency (SOL), and the proportions of light sleep (N1) and deep sleep (N3). Sunlight exposure was defined as the number of hours above 1000 lux during daytime, and artificial light and smartphone use before bedtime were quantified as the duration of exposure accumulated in the 2 hours preceding sleep onset. Linear mixed-effects models with a random intercept at the individual level estimated the associations between these exposures and next-night sleep outcomes, adjusting for step count and other individual covariates.

**Results:**

The average TST was 420 (SD 85) minutes, and SOL averaged 17.6 (SD 18) minutes. Light sleep (N1) represented 6.6% (SD 2.1%) of sleep, and deep sleep (N3) accounted for 20.1% (SD 7.6%). Each additional hour of daytime sunlight exposure was associated with an increase of 10.67 (95% CI 0.6-20.7) minutes in TST the following night and with a 0.3 (95% CI –0.6 to –0.0) percentage-point decrease in light sleep (N1) percentage. No associations were found between evening artificial light exposure and sleep outcomes, while each minute of smartphone use before bedtime was linked to an increase in SOL of 0.2 (95% CI 0.0-0.4) minutes.

**Conclusions:**

These findings emphasize the importance of daylight exposure for circadian alignment and the potential sleep-disruptive effects of evening digital engagement. This study demonstrates the feasibility and value of integrating wearable electroencephalography and environmental and behavioral sensors in naturalistic settings to uncover behavioral and environmental correlates of sleep architecture.

## Introduction

### Sleep Dynamics

Sleep is a fundamental biological process that is crucial for maintaining overall health and well-being, playing a vital role in cognitive function, emotional regulation, metabolism, and immune system performance [1]. Insufficient or poor-quality sleep has been associated with a wide range of health issues, including cardiovascular and metabolic dysfunction, mental health disorders, cognitive decline, and increased all-cause mortality [2-5].

Sleep is a dynamic process made up of distinct stages, which collectively determine its architecture. Sleep is generally divided into non–rapid eye movement sleep, which includes light (stage 1 sleep [N1] and stage 2 sleep [N2]) and deep sleep (stage 3 sleep [N3]), and rapid eye movement (REM) sleep [6]. Each sleep stage serves critical functions: light sleep allows transitions from wakefulness into sleep and subsequent sleep stages, deep sleep supports physiological restoration and recovery, and REM sleep contributes to cognitive processing and emotional regulation [7,8]. Sleep occurs in ultradian cycles that alternate between non–rapid eye movement and REM sleep approximately every 90 minutes [9]. The first cycles are characterized by a higher prevalence of deep sleep, while REM sleep becomes increasingly prominent in the later cycles [6]. These cyclical processes are regulated by circadian rhythms of the body’s internal clock, which synchronizes sleep timing with environmental cues such as light and darkness [10].

### Circadian Entrainment and Disruption

Circadian entrainment refers to the alignment of the internal circadian clock with external environmental cues, such as the light-dark cycle of the day. Sunlight is the most powerful entrainer, as it modulates the release of melatonin, a hormone secreted by the pineal gland that regulates the timing of sleep and wakefulness [10]. During daylight, sunlight suppresses melatonin secretion, promoting wakefulness and alertness; as light diminishes in the evening, melatonin levels rise, facilitating sleep onset [11]. Adequate daytime sunlight exposure therefore strengthens circadian entrainment, leading to earlier sleep onset and longer total sleep time (TST) [12]. Consistent evidence shows that more time spent outdoors, exposed to natural light, is associated with a stronger circadian rhythm and better mood and sleep outcomes [13,14].

Conversely, circadian disruption occurs when external factors or individual behaviors cause misalignment between endogenous circadian rhythms and the external environment [15]. This misalignment may also disrupt the synchronization between the body’s central clock, located in the suprachiasmatic nucleus, and peripheral clocks in tissues and organs, potentially contributing to dysregulation of physiological and behavioral rhythms such as hormone release, metabolism, and the sleep-wake cycle [10,15]. A key contributor to circadian disruption is artificial light at night, particularly the blue light emitted from digital screens and smartphones. This stimulation suppresses melatonin production, which normally supports circadian alignment and promotes sleep propensity, thereby delaying sleep onset and altering sleep architecture [11,16].

Behavioral circadian modulators further influence the alignment or misalignment of circadian rhythms. In addition to blue light exposure, smartphone use before bedtime also provides stimulating content (eg, social media) that may increase cognitive and physiological arousal, activating the hypothalamic-pituitary-adrenal axis and elevating cortisol levels, ultimately interfering with sleep initiation and maintenance [17]. However, there is limited and mixed evidence regarding how and to what extent smartphone use might affect sleep stages, and thus, the topic warrants further investigation [18].

Engaging in regular physical activity, particularly earlier in the day, can promote circadian entrainment by reinforcing the distinction between active daytime and restful nighttime periods [19-21]. Exercise has been associated with improved sleep onset and increased proportions of deep sleep (N3), whereas intense physical activity close to bedtime may elevate core body temperature and arousal levels, potentially delaying sleep onset and disrupting sleep architecture by reducing REM sleep and increasing light sleep [21,22]. Other health-related behaviors, such as napping, evening food intake, and the consumption of alcohol, caffeine, or medications, can also affect sleep and circadian rhythms by altering metabolism, arousal and sleep pressure, delaying sleep onset, and decreasing deep and REM sleep [23-25].

### Gaps in the Literature and Rationale of the Study

Despite the recognized role of environmental and behavioral factors in sleep and circadian regulation, there are gaps in the literature concerning their combined effects on sleep architecture. Regarding the variables examined in the literature, previous studies have mainly focused on either sunlight exposure or artificial light at night in isolation, without considering the additional influence of other behavioral modulators such as smartphone use and physical activity [13,14,26,27]. For instance, time spent outdoors often correlates with higher physical activity, which also impacts sleep [28,29]. Controlling for physical activity is therefore essential when assessing the effects of sunlight exposure on sleep outcomes to avoid confounding effects.

Regarding sleep and circadian health, an increasing number of studies are adopting sensor-based methods to measure sleep and physical activity in both observational studies and clinical trials [30,31]. However, the majority of these studies rely on accelerometry to assess sleep outcomes, which, while useful for estimating general sleep metrics (eg, TST and sleep efficiency [SE]), lacks the ability to provide detailed insights into its architecture and sleep stages [32-36]. There is a scarcity of research using wearable electroencephalography devices in real-life settings outside controlled experimental environments, which offer more precise measurements of sleep stages compared to other sensors [32,37,38]. Finally, regarding study designs, only a minority of these studies have used longitudinal frameworks with repeated measures to capture the day-to-day variability in environmental exposures, behaviors, and sleep outcomes over multiple days of tracking [39,40]. In contrast, the majority of studies have adopted cross-sectional designs, limiting their ability to move beyond mere associations and understand causal relationships through assessment of within-person processes [39,40].

### Objectives

Our study addresses gaps in the literature by conducting a 7-day sensor-based investigation assessing sunlight exposure, artificial light at night, and behavioral circadian modulators. Through continuous light monitoring, smartphone use tracking, and physical activity measurement, we aim to comprehensively evaluate the effects of circadian entrainment and disruption on electroencephalography-based sleep metrics. This research not only fills a critical gap in the existing literature but also has practical implications for developing interventions and guidelines to enhance sleep health through the management of light exposure and sleep-hygiene behavioral practices.

In this study, we selected a priori TST, sleep onset latency (SOL), light sleep (N1), and deep sleep (N3) as main sleep outcomes, based on their theoretical relevance to sleep and circadian health. TST is the most used sleep measure in the literature and reflects overall sleep duration [38]. SOL measures the time taken to fall asleep, reflecting the ease of initiating sleep and showing sensitivity to presleep behaviors that may delay sleep onset [41]. N1 marks the transition from wakefulness to deeper sleep stages in each cycle and serves as an indirect marker of sleep fragmentation and efficiency [42]. N3, the most restorative sleep stage for physical health, responds strongly to daily activity and circadian alignment, making it valuable for evaluating how behavioral factors affect sleep [19,42].

First, we aim to quantify the effect of sunlight exposure during the day, as a proxy for circadian entrainment, on sleep. Second, we seek to measure the impact of potential circadian disruptors, such as exposure before bedtime to bright artificial light and smartphone use, on sleep. We hypothesize that (1) longer sunlight exposure will be associated with longer TST, a lower percentage of light sleep (N1), and a higher percentage of deep sleep (N3) and (2) exposure to bright artificial light and smartphone use close to bedtime will be associated with shorter TST, increased SOL, a higher proportion of light sleep (N1), and a lower percentage of deep sleep (N3).

## Methods

### Participants and Study Design

Our study included 21 participants from the Jerusalem metropolitan area (Israel) and was conducted from May 2023 to July 2024. Participants were monitored over a 7-day period using wearable sensors and smartphone apps.

Given the small sample size, a quota sampling approach was adopted to recruit participants for this study, allowing for diversity and representation across key demographic groups (ie, household income, age, and gender) without requiring strict proportionality to the broader population.

Recruitment was conducted in 2 stages: first, an Israeli panel company contacted individuals from their preestablished pool of volunteers who had previously signed up for scientific studies. The panel company confirmed participants’ willingness to participate and screened them for eligibility through a questionnaire based on the study’s recruitment criteria. The main inclusion criteria required participants to be aged between 18 and 65 years and to reside in the metropolitan area of Jerusalem. Exclusion criteria included engaging in any form of shift work, having mobility limitations, significant health conditions, or a clinical diagnosis of sleep or mental health disorders. In the second stage, a research assistant followed up with eligible candidates by phone to explain the study in greater detail, further inviting them to participate and initiating the scheduling of data collection.

Following initial sampling, eligible participants were onboarded through a home visit conducted by a research assistant, marking the start of the data collection period. During this visit, the research assistant administered an entry online questionnaire collecting key demographic information and explained the study protocol, emphasizing the significance of their involvement in studying urban health. Participants received an information sheet outlining the sensor charging and wearing schedule, along with a contact number for questions and support.

### Ethical Considerations

The study received ethical approval from The Hebrew University of Jerusalem’s Committee for the Use of Human Subjects in Research (approval number 22022023; February 2023). All participants provided informed consent prior to participation and could withdraw at any time without consequence. The study adhered to the General Data Protection Regulation and institutional ethical standards for human research, ensuring compliance with data storage, sharing, and confidentiality requirements. All collected data were pseudonymized before analysis, and identifiable information was stored separately in secure, password-protected databases. Upon successful completion of the 7-day protocol, participants were compensated with ILS 300 (US $104) as an acknowledgement of their cooperation and motivation to adhere to the study.

### Sensor-Based and Mobile Measures

#### Sleep Assessment

**Figure 1:**
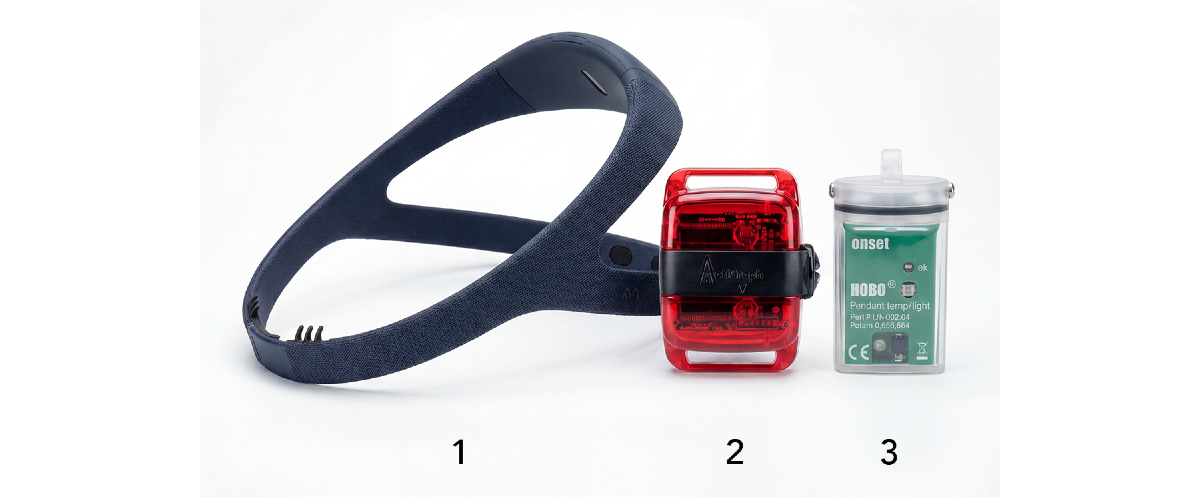
Study Sensors: (1) Dreem 3 Headband (Beacon Biosignals); (2) wGT3X+ triaxial accelerometer (ActiGraph); (3) HOBO Data Logger (Onset Computer Corporation).

Each night, participants’ TST, SE, SOL, wake after sleep onset, and the duration of sleep stages (N1, N2, N3, and REM) were assessed using the Dreem Headband, a wearable electroencephalography device (Figure 1). The headband has been validated against polysomnography, the gold standard for sleep measurement, demonstrating high accuracy in sleep staging when compared to manual scoring by sleep experts [32]. This nonintrusive device provides reliable sleep assessment in real-world, home settings, providing an advantage over actigraphy devices, which are unable to differentiate sleep stages accurately [32,35,36].

#### Light Exposure

Participants wore a HOBO Data Logger daily, attached to the collar of their clothing with a safety pin. This positioning was intended to approximate light exposure at the level of the eyes, where light signals are transmitted from the retina to the suprachiasmatic nucleus, the central circadian pacemaker [33]. The timing, duration, and intensity of light exposure were used to assess participants’ daily circadian entrainment to sunlight and potential disruption from artificial light at night. Based on previous research, daytime sunlight exposure was operationalized as the total duration (in hours) of exposure to light intensity above 1000 lux across the day [43]. Before bedtime, artificial light exposure was defined as the total duration (in minutes) of exposure to light intensity above 30 lux across varying time windows in the 2 hours preceding sleep onset.

#### Physical Activity

Participants carried a wGT3X+ triaxial accelerometer on a waist belt on the right hip every day during waking hours. The accelerometer was used to assess various physical activity metrics, including step count (in units of 1000 steps), metabolic expenditure, time spent in moderate to vigorous physical activity, light physical activity, and sedentary time. Nonwear periods were identified and excluded using the Choi algorithm [44]. Physical activity metrics were calculated using established criteria, such as the Freedson Adult VM3 cut-points (2011) and the Crouter 2R regression equation [45,46]. After preliminary analyses, we selected step count over the other physical activity metrics for its greater explanatory power in capturing sleep outcome variability.

#### Smartphone Usage Tracking App

A designated app installed on participants’ smartphones tracked the total time spent using the smartphone and the time spent using apps for the whole data collection period. The app was developed at the Department of Geography and the Center for Urban Innovation at the Hebrew University of Jerusalem. For analysis, apps were classified into categories (eg, social media, productivity, and entertainment), and the original app names were removed to ensure participant privacy. From these reports, we computed for each day the total number of minutes spent using each app category. However, preliminary analyses revealed no meaningful differences between total smartphone use and the use of social media apps in explaining sleep outcomes variability. Therefore, for our main analyses, we used total smartphone use, as it encompasses all types of smartphone activity, offering a more inclusive measure.

### Statistical Methods

We used linear mixed-effects models with a random intercept at the individual level to examine the effects of circadian entrainment and disruption on sleep outcomes. This approach accounted for the nested hierarchical structure of the data (successive nights nested within participants) and controlled for between-subject variability in baseline sleep patterns.

The outcomes modeled were TST, percentages of N1 and N3, and SOL. To test the first hypothesis, that higher daytime sunlight exposure is associated with better sleep outcomes, we used daily sunlight exposure (measured in hours) as the primary predictor, with daily step count as a covariate to control for potential confounding from physical activity. To test the second hypothesis, we examined whether exposure to artificial light and smartphone use before bedtime negatively affect sleep outcomes. The primary predictors were artificial light exposure (measured as minutes of exposure above 30 lux [ie, dim light], a physiologically relevant threshold used in previous protocols [47]) and smartphone use (in minutes) in the 2 hours before bedtime. Step count in the same time window was included as a covariate to control for potential confounding from physical activity. Before selecting the 2-hour window for artificial light, we conducted a sensitivity analysis across different time windows before bedtime (5, 10, 15, 20, 25, 30, 60, and 120 minutes) to assess potential differences in their associations with sleep outcomes. As no meaningful differences were detected across these intervals, we proceeded with the most inclusive duration of 2 hours before bedtime to capture potential cumulative effects and maximize variability.

For covariate selection, we initially considered a broad set of potential confounders, refining the final model based on preliminary analyses (Table 1). The final selected covariates for all models included age group (25-29 years and 30-65 years), gender (female and male), household income per unit of consumption (low: ILS ≤5550 [US $1900] and high: ILS >5550 [US $1900]), weekday vs weekend, and season (spring, summer, autumn, and winter) to account for possible seasonal influences on sleep and light exposure.

**Table 1:** Participants’ characteristics^a^.

Characteristics		Values, n (%)
**Gender**		
	Female	14 (67)
	Male	7 (33)
**Age (years)**		
	25-29	10 (48)
	30-65	11 (52)
**Education**		
	University	9 (43)
	Professional	4 (19)
	High school	8 (38)
**Employment**		
	Unemployed	3 (14)
	Employed	18 (86)
**Household income (ILS 1=US $0.34)**		
	High (>7800)	4 (19)
	Medium (5551-7800)	5 (24)
	Low (≤5550)	12 (57)
**Children**		
	No	13 (67)
	Yes	7 (33)
**Bed partner**		
	No	12 (62)
	Yes	8 (38)
**Citizenship**		
	Non-Israeli citizen	5 (24)
	Israeli citizen	16 (76)
**BMI (kg/m^2^)**		
	Normal	11 (52)
	Overweight	10 (48)
**Neighborhood**		
	Suburban area	12 (67)
	City center	7 (33)

^a^N=21.

Due to constraints in data availability, we tested separate models for our 2 hypotheses rather than combining all the predictors into one comprehensive model. Including all predictors in a single model would have reduced the sample size, as observations with missing data on any predictor would have been excluded, affecting model power and stability. By analyzing these factors in independent models, we maximized the use of available data to evaluate each predictor’s relationship with sleep outcomes. No adjustment for multiple comparisons was performed, as models were specified a priori and focused on a limited set of exposures and outcomes. Findings are therefore interpreted cautiously in light of the modest sample size and correlated predictors and outcomes. Statistical analyses were conducted using the R software (version 4.0.2; R Core Team; 2017).

## Results

### Descriptive Statistics

Of the 21 participants, 14 (67%) were female, and 11 (52%) were aged between 30 and 65 years; 16 (76%) were Israeli citizens, 9 (43%) had university education, and 12 (57%) reported a lower household income (ILS ≤5550 [US $1900]). See Table 1 for additional details.

The average TST was 420 (SD 85.4) minutes per night, ranging from 120 to 667.5 minutes across specific nights among participants. SOL averaged 17.6 (SD 18) minutes, with a range of 0.5-99 minutes across specific nights. N1 sleep accounted, on average, for 6.6% (SD 2.1%) of TST, ranging from 2.6% to 13.1%. N3 sleep reached, on average, 20.1% (SD 7.6%) of TST, with a range of 5.2%-51.1% across specific nights. See Table 2 for more sleep outcomes.

**Table 2:** Descriptive statistics on sleep outcomes.

Outcome	Mean (SD)	Range
TST^a^ (min)	420 (85.4)	120-667.5
WASO^b^ (min)	29.1 (25)	1-143
Sleep efficiency (%)	89.4 (6.7)	66.2-97.9
Sleep onset latency (min)	17.6 (18)	0.5-99
N1^c^ (%)	6.6 (2.1)	2.6-13.1
N2^d^ (%)	48 (7.6)	22.1-65.6
N3^e^ (%)	20.1 (7.6)	5.2-51.1
REM^f^ (%)	25.4 (7.9)	1.4-57.6

^a^TST: total sleep time.

^b^WASO: wake after sleep onset.

^c^N1: stage 1 sleep.

^d^N2: stage 2 sleep.

^e^N3: stage 3 sleep.

^f^REM: rapid eye movement sleep.

Participants spent an average of 1.2 (SD 1.6) hours exposed to sunlight, with a range from 0 to 8.3 hours across specific days across participants. Time spent using smartphone apps was 169 (SD 178) minutes per day on average, with social apps accounting for 44.0% (SD 21.9%) of that time. Daily step count averaged 6033 (SD 3779), ranging from 57 to 15,516 steps across specific days. See Table 3 for further details.

**Table 3:** Descriptive statistics on the main predictors.

Predictor	Mean (SD)	Range
Daily sunlight^a^ (hours)	1.2 (1.6)	0-8.3
Before bedtime artificial light^b^ (minutes)	20.9 (33.7)	0-120
Daily smartphone use^c^ (minutes)	169.7 (176.9)	0-661.1
Before bedtime smartphone use^d^ (minutes)	20.3 (25.2)	0-105.7
Daily step count^e^ (1000 steps)	6.3 (3.9)	0.1-15.5
Before bedtime step count^f^ (1000 steps)	0.6 (0.8)	0-3.8

^a^Daily sunlight: time spent exposed to light above 1000 lux during daytime hours (hours).

^b^Before bedtime artificial light: time spent exposed to light above 30 lux in the 2 hours preceding sleep onset (minutes).

^c^Daily smartphone use: total daily duration of smartphone activity (minutes).

^d^Before bedtime smartphone use: total duration of smartphone activity in the 2 hours preceding sleep onset (minutes).

^e^Daily step count: number of steps across the day (expressed in thousands).

^f^Before bedtime step count: number of steps in the 2 hours preceding sleep onset (expressed in thousands).

### Sunlight Exposure and Sleep

In this study, the association between daily sunlight exposure (in hours) and key sleep outcomes the following night was analyzed, as shown in Table 4. Each additional hour of sunlight exposure was associated with an estimated increase of 10.7 (95% CI 0.6-20.7) minutes in TST. In terms of sleep architecture, sunlight exposure was associated with a decrease in the percentage of N1 sleep by 0.3 (95% CI –0.6 to –0.0) percentage points, while no association with N3 sleep was detected.

**Table 4:** Associations between sunlight exposure and sleep outcomes (116 observations; 21 participants).

			TST^a^ (min)	N1^b^ (%)	N3^c^ (%)
**Predictors, estimate^d^ (95% CI)**					
	Intercept		410.58 (335.15 to 486.00)	6.41 (3.77 to 9.05)	18.14 (9.39 to 26.88)
	Daily sunlight^e^		10.67 (0.64 to 20.70)	–0.29 (–0.55 to –0.03)	–0.45 (–1.49 to 0.59)
	Age (30-65 years)		–53.43 (–121.57 to 14.70)	–0.79 (–3.20 to 1.61)	0.10 (–7.84 to 8.03)
	Gender (male)		–28.24 (–97.45 to 40.96)	0.81 (–1.64 to 3.26)	0.68 (–7.39 to 8.75)
	**Season (vs autumn)**				
		Spring	23.35 (–59.25 to 105.94)	–1.05 (–3.86 to 1.76)	1.89 (–7.59 to 11.37)
		Summer	62.64 (–9.12 to 134.40)	–0.23 (–2.76 to 2.31)	0.93 (–7.43 to 9.30)
		Winter	70.37 (–31.98 to 172.72)	–0.42 (–4.02 to 3.18)	–1.34 (–13.24 to 10.56)
	Weekend (vs weekday)		–2.98 (–34.47 to 28.52)	0.05 (–0.73 to 0.82)	1.71 (–1.47 to 4.89)
	Household income (high)^f^		7.83 (–50.34 to 66.01)	1.00 (–0.98 to 2.98)	3.17 (–3.51 to 9.85)
	Bed partners (yes)		–33.41 (–101.64 to 34.81)	1.47 (–0.89 to 3.83)	–0.49 (–8.37 to 7.38)
**Other information**					
	σ^2g^		4457.95	2.66	45.18
	τ00 ID^h^		1743.20	2.72	26.48
	ICC^i^		0.28	0.51	0.37
	Marginal *R*^2^/conditional *R*^2^		0.11/0.36	0.11/0.56	0.06/0.41

^a^TST: total sleep time.

^b^N1: stage 1 sleep.

^c^N3: stage 3 sleep.

^d^Estimate of effect size.

^e^Daily sunlight: hours of exposure to light above 1000 lux.

^f^Household income (ILS): high (>5551); low (≤5550); ILS 1=US $0.34.

^g^σ^2^: residual variance.

^h^τ00 ID: variance between individuals.

^i^ICC: intraclass correlation coefficient (variance attributed to individual differences).

A separate model was estimated to adjust for physical activity, measured as daily step count (per 1000 steps). This adjustment reduced the analytic sample by 40 observations, including all data from 2 participants, warranting separate estimation. In this model, the association between sunlight exposure and TST was unchanged (each additional hour of sunlight being associated with an increase of 11.6 (95% CI –0.5 to 23.8) minutes in TST, although the CI included 0. In contrast, higher daily step count was associated with longer TST, with an estimated increase of 6.1 (95% CI 1.0-11.1) minutes per additional 1000 steps. Further details are provided in Multimedia Appendix 1.

### Circadian Disruptors and Sleep

In examining the impact of circadian disruption on sleep outcomes, we evaluated the associations of artificial light exposure, step count, and smartphone use recorded in the 2 hours prior to bedtime with key sleep outcomes the following night, as shown in Table 5. While no associations were observed between artificial light exposure or step count and TST, N1, or N3 sleep stages, smartphone use showed an association with SOL, where each additional minute of smartphone use before bedtime was associated with an increase in SOL by 0.2 (95% CI 0.0-0.4) minutes.

**Table 5:** Associations of artificial light, step count, and smartphone use before bedtime with sleep outcomes (75 observations; 18 participants).

			TST^a^ (min)		SOL^b^		N1^c^ (%)		N3^d^ (%)	
**Predictors, estimate^e^ (95% CI)**										
	Intercept		348.06 (235.39 to 460.72)		32.04 (8.19 to 55.88)		4.54 (1.46 to 7.63)		20.60 (8.80 to 32.41)	
	Before bedtime smartphone use^f^		0.15 (–0.74 to 1.04)		0.20 (0.00 to 0.39)		0.01 (–0.01 to 0.03)		–0.04 (–0.12 to 0.03)	
	Before bedtime artificial light^g^		0.26 (–0.69 to 1.21)		0.02 (–0.19 to 0.23)		0.01 (–0.01 to 0.04)		–0.00 (–0.09 to 0.08)	
	Before bedtime step count^h^		–0.01 (–0.03 to 0.02)		0.00 (–0.00 to 0.01)		0.00 (–0.00 to 0.00)		0.00 (–0.00 to 0.00)	
	Age (30-65 years)		–49.53 (–139.82 to 40.76)		2.64 (–16.41 to 21.70)		–0.21 (–2.70 to 2.28)		0.82 (–8.76 to 10.40)	
	Gender (male)		4.57 (–84.54 to 93.67)		–14.97 (–33.74 to 3.80)		1.06 (–1.41 to 3.52)		–0.84 (–10.37 to 8.69)	
	**Season (vs autumn)**									
		Spring		137.13 (26.90 to 247.35)		–12.68 (–36.09 to 10.73)		–0.68 (–3.67 to 2.32)		–0.03 (–11.36 to 11.31)
		Summer		108.31 (14.69 to 201.93)		–15.03 (–34.84 to 4.78)		0.31 (–2.25 to 2.88)		–1.28 (–11.10 to 8.54)
		Winter		136.27 (9.96 to 262.57)		6.68 (–19.99 to 33.36)		–0.23 (–3.71 to 3.24)		–0.72 (–14.08 to 12.64)
	Weekend (vs weekday)		–19.78 (–67.62 to 28.05)		7.03 (–3.58 to 17.65)		0.45 (–0.73 to 1.63)		–0.44 (–4.38 to 3.50)	
	Household income (high)^i^		–12.35 (–84.25 to 59.56)		–0.66 (–15.91 to 14.58)		1.40 (–0.56 to 3.36)		0.75 (–6.71 to 8.21)	
	Bed partners (yes)		–17.25 (–104.18 to 69.67)		–21.44 (–39.75 to –3.13)		2.35 (–0.05 to 4.75)		–3.96 (–13.25 to 5.33)	
**Other Information**										
	σ^2j^		5045.09		249.22		3.05		33.68	
	τ00 ID^k^		1933.94		79.14		1.69		28.56	
	ICC^l^		0.28		0.24		0.36		0.46	
	Marginal *R*^2^/conditional *R*^2^		0.18/0.41		0.36/0.50		0.19/0.48		0.09/0.51	

^a^TST: total sleep time.

^b^SOL: sleep onset latency.

^c^N1: stage 1 sleep.

^d^N3: stage 3 sleep.

^e^Estimate of effect size.

^f^Before bedtime smartphone use: minutes of smartphone use in the 120 minutes before sleep time.

^g^Before bedtime artificial light: minutes of exposure to light above 30 lux in the 120 minutes before sleep time.

^h^Before bedtime step count: number of steps in the 120 minutes before sleep time (expressed in thousands).

^i^Household income (ILS): high (>5551); low (≤5550); ILS 1=US $0.34.

^j^σ^2^: Residual variance.

^k^τ00 ID: variance between individuals.

^l^ICC: intraclass correlation coefficient (variance attributed to individual differences).

## Discussion

### Principal Findings

Our findings support the first hypothesis that higher daytime exposure to sunlight is associated with increased TST and a reduction in light sleep (N1). Specifically, each additional hour of sunlight exposure was linked to an increase of approximately 10.7 (95% CI 0.6-20.7) minutes in TST and a decrease of 0.3 (95% CI –0.6 to –0.0) percentage points in N1 sleep. These results align with previous research suggesting that natural light exposure strengthens circadian entrainment, leading to improved sleep duration and quality [12-14]. Similarly, the potential circadian entrainment from increased sunlight exposure might have contributed to reduced N1, suggesting a quicker transition from wakefulness into deeper sleep stages [42,48]. This reduction in N1 could reflect enhanced circadian alignment, promoting a smoother progression into the other sleep stages. However, we did not observe an association between sunlight exposure and deep sleep (N3), which contrasts with our initial hypothesis. While some studies have reported that natural light exposure can enhance deep sleep [49], our results suggest that the relationship may be more nuanced. It is possible that factors such as individual differences in circadian sensitivity, genetic predispositions, or, most importantly, the timing of sunlight exposure might play a role [49-51]. The effect of sunlight exposure on N3 might be more pronounced when it occurs shortly after wake-up time, when the sun’s blue-enriched morning light exerts stronger phase-advancing effects on circadian entrainment [51,52]. Using a daily cumulative indicator of sunlight exposure could average out these timing-specific effects, potentially obscuring its impact on deep sleep, which should be further examined in future sensor-based research.

After controlling for daily step count, the point estimate of the association between sunlight exposure and TST was even slightly increased, suggesting the absence of substantial confounding by physical activity. The CI of the association included 0 in this last model, likely due to reduced statistical power from the smaller sample of 76 observations with both sunlight and physical activity data, compared to 116 in the previous model. Increased physical activity during the day might have extended TST by promoting a greater need for physiological recovery, enhancing overall sleep drive [53]. On the other hand, the independent association between sunlight exposure and a decrease in N1 sleep remained fully apparent in the model controlling for step count with fewer observations, highlighting the unique role of natural light in regulating sleep architecture beyond physical activity levels. This finding is noteworthy because it isolates the effect of sunlight from major confounders like physical activity, which is often correlated with time spent outdoors [28,29]. Additionally, this pattern suggests that physical activity may not mediate the relationship between sunlight exposure and improved sleep architecture, although a formal mediation analysis would be warranted to confirm this interpretation and to clarify the interplay between sunlight exposure, physical activity, and sleep. Remarkably, daily step count, used as a proxy for physical activity, was not associated with N1 or N3 percentages, despite evidence that higher levels of physical activity generally enhance SE and promote more restorative sleep with reduced light sleep and increased deep sleep [19,21,53].

The positive associations between daytime sunlight exposure, physical activity, and better sleep outcomes underscore the importance of natural light and movement for supporting circadian alignment and sleep health. Encouraging daily exposure to natural light and regular physical activity, individually or in combination, could be an effective strategy for improving sleep quality across the general population. These findings support public health recommendations encouraging time outdoors and physical activity to enhance sleep and circadian health, ultimately fostering overall well-being.

Contrary to our second hypothesis, we did not find any association between exposure to artificial light before bedtime and alterations in TST, SOL, or sleep architecture. This finding diverges from prior studies that have reported negative impacts of evening exposure to artificial light on sleep onset, duration, and stages with increases in light sleep and reductions in deep sleep [16,26]. This discrepancy may be due to the relatively low intensity or limited duration of artificial light exposure in our sample, which may have been insufficient to affect sleep architecture. Additionally, individual differences in light sensitivity and circadian preference may influence susceptibility to artificial light’s effects on sleep [54]. Furthermore, participants may have exhibited overall good sleep hygiene, with the cumulative benefits of various positive behaviors potentially offsetting any negative impact of artificial light on sleep [55]. Finally, it is also possible that the light sensor was incorrectly worn by some of the participants during the hours just before sleep time.

Regarding smartphone use, while no associations were observed with TST, N1, or N3 sleep stages, an association with increased SOL was detected, indicating that smartphone use before bedtime may delay sleep onset. Since our model controlled for artificial light exposure, this delay likely stems not from light emitted by screens but rather from the arousal-increasing and mentally engaging nature of smartphone activities. This aligns with evidence that smartphone use before bedtime can increase cognitive arousal and hinder the presleep relaxation process critical for facilitating sleep onset [56].

The mixed findings regarding artificial light and smartphone use highlight the complexity of assessing individual sleep behaviors and their impacts. The lack of observed negative effects from artificial light before bedtime could indicate that its impact on sleep may be less pronounced or more individualized than previously thought, underscoring the need for an idiographic approach in future research to capture individual variations in light sensitivity [57]. The association between smartphone use and increased SOL suggests that smartphone engagement before bedtime can interfere with the winding-down process necessary for sleep. This points to the importance of limiting mentally stimulating activities in the evening to promote a smoother transition to sleep. Our findings support the inclusion of recommendations in sleep hygiene guidelines to reduce evening smartphone use as a strategy to maximize sleep and circadian health.

### Strengths and Limitations of the Study

A major strength of this study is the use of innovative sensor-based tools to collect objective, high-resolution data on sleep architecture, light exposure, physical activity, and smartphone use in real-world settings. The use of wearable electroencephalography devices provided precise measurements of sleep stages, offering a significant advantage over traditional actigraphy, which cannot accurately differentiate between sleep stages, and an even greater advantage over self-reported sleep assessments via questionnaires commonly used in studies on the environmental determinants of sleep [32]. The continuous monitoring of light exposure using data loggers positioned near eye level allowed for accurate estimation of circadian entrainment and disruption factors. Additionally, the integration of a smartphone app to track usage patterns provided insights into a major behavioral circadian disruptor. The repeated measures design over 7 consecutive days enabled us to capture day-to-day variability and to better identify individual differences, offering a nuanced view of how environmental exposures and behaviors impact sleep architecture over time.

However, the study has several limitations. The short duration and small sample size of the study may limit the generalizability of the findings. While we accounted for seasonal variation, the 7-day monitoring period may have restricted our capacity to fully capture its influence on sleep patterns [39]. Additionally, the limited sample size prevented us from controlling for sleep autocorrelation, a key component of sleep variability [39,57], and may have limited our ability to detect meaningful associations. Future studies with extended monitoring periods and larger samples are needed to account for temporal dynamics and enhance precision in understanding sleep variability and its determinants.

Due to missing data and limited observations, we were also unable to include all relevant predictors in a single comprehensive model, which restricted our ability to evaluate potential reciprocal confounding effects between the exposures. Furthermore, although smartphone ecological momentary assessments captured several health-related behaviors in our study, excessive missing data prevented us from using these measures to account for behaviors with known disruptive effects on sleep, such as the timing of caffeine, alcohol, medication, and food intake, napping habits, or the use of blue light–blocking lenses or screen filters [23-25]. These unmeasured confounders could have influenced the associations that we observed or our ability to identify associations that we were unable to detect. Finally, the reliance on light intensity measurements without considering the spectral composition of light limited our ability to differentiate between the effects of different light wavelengths on circadian rhythms [16,51]. Including such variables in future studies could improve the accuracy and comprehensiveness of the findings.

Despite these limitations, our study highlights the potential of sensor-based methods in advancing sleep research. By demonstrating the feasibility of integrating multiple wearable devices and a smartphone app to capture detailed environmental and behavioral data, we contribute to the development of ecologically valid approaches to studying sleep and circadian health. Serving as a proof of concept, this approach lays the groundwork for future studies with larger samples and extended monitoring periods, paving the way for more ecologically valid investigations into the complex interactions between environmental exposures, behaviors, and sleep outcomes.

### Conclusions

Our findings underscore the beneficial impact of daily sunlight exposure and physical activity on enhancing sleep duration and reducing light sleep, highlighting the importance of natural light and movement for circadian alignment and overall sleep health. While artificial light before bedtime showed no association with sleep, smartphone use was linked to delayed sleep onset, potentially due to its stimulating effects. These results support public health recommendations to encourage outdoor exposure to sunlight and more physical activity while advising limited smartphone use in the evening to improve sleep and circadian health. These insights contribute to a better understanding of the factors influencing sleep architecture and underscore the potential of sensor-based methodologies in advancing sleep research.

## Appendix

Multimedia Appendix 1 Details of the model.

## Abbreviations

N1: stage 1 sleep
N2: stage 2 sleep
N3: stage 3 sleep
REM: rapid eye movement
SE: sleep efficiency
SOL: sleep onset latency
TST: total sleep time

## Data Availability

The dataset analyzed in this study is available from the corresponding author upon request.
