# Supplementary material for: The Impact of Sunlight and Artificial Light at Night on Sleep Stages: Evidence From a 7-Day Sensor-Based Observational Study
Source: JMIR Mhealth Uhealth. 2026 Jun 5;14:e75898. doi: 10.2196/75898 (PMC13282595; doi:10.2196/75898)
Supplement: Multimedia Appendix 1 [file mhealth_v14i1e75898_app1.docx]

# Circadian entrainment and circadian disruption: the impact of sunlight and artificial light at night on sleep stages in a 7-day sensor-based study

Andrea Montanari,^1^ Limin Wang^2^, Amit Birenboim ^2,3^, Basile Chaix^1^.

*^1^ Sorbonne Université, INSERM, Institut Pierre Louis d’Epidémiologie et de Santé Publique IPLESP, Nemesis team, Faculté de Médecine Saint-Antoine, 27 rue Chaligny, 75012 Paris, France.*

*^2^ The Hebrew University of Jerusalem, Department of Geography, Mt Scopus, Jerusalem, 919051 Israel.*

*^3^ The Hebrew University of Jerusalem, Department of Geography and The Center for Urban Innovation, Mt Scopus, Jerusalem, 919051 Israel.*

*Correspondance:* [*andrea.montanari@iplesp.upmc.fr*](mailto:andrea.montanari@iplesp.upmc.fr)

# Supplemental Material: Appendices

## Appendix I

### Methods

**Table 1***Summary of model specifications and goals*

| **Family of Models** | **Table** | **Observations / Participants** | **Formula** |
| --- | --- | --- | --- |
| 1 - Associations of daily sunlight exposure with sleep outcomes | Table 4  (Manuscript) | 116 / 21 | *lmer(TST ~ (1 \| participant ID) + daily_sunlight_exposure + age_binary + gender + season + weekend + household_income_binary + bed_partner + data = surreal)* |
| 2- Associations of before bedtime artificial light, step count and smartphone use with sleep outcomes | Table 5 (Manuscript) | 75 / 18 | *lmer(TST ~ (1 \| participant ID) + bedtime_smartphone_use + bedtime_light_exposure +bedtime_step_count + age_binary + gender + season + weekend + household_income_binary + bed_partner + data = surreal)* |
| 3 - Associations of daily sunlight exposure and step count with sleep outcomes | Table 6 (Appendices) | 76 / 19 | *lmer(TST ~ (1 \| participant ID) + daily_sunlight_exposure + daily_step_count + age_binary + gender + season + weekend + household_income_binary + bed_partner + data = surreal)* |

*Every family of models tested the following outcomes: total sleep time (TST), sleep onset latency (SOL), sleep stage 1 (N1), sleep stage 3 (N3)*

## Appendix II

### Results

#### Descriptive Statistics

Table 2 summarizes the number of valid sleep observations collected for each participant across the seven monitoring days. Each row represents an individual participant (ID), showing the total number of nights with valid electroencephalography-derived sleep recordings included in the analysis. On average, 5.6 nights per participant were available, totaling 117 observations. Please refer to Table 2 for a detailed overview of sleep observations per participant. **Table 2**

*Sleep observation per participant*

| **ID Participant** | **Sleep  Observations** |
| --- | --- |
| 1 | 7 |
| 2 | 8 |
| 3 | 7 |
| 4 | 7 |
| 5 | 7 |
| 6 | 6 |
| 7 | 4 |
| 8 | 6 |
| 11 | 4 |
| 12 | 7 |
| 13 | 7 |
| 14 | 5 |
| 16 | 3 |
| 17 | 4 |
| 18 | 5 |
| 19 | 4 |
| 21 | 7 |
| 22 | 7 |
| 23 | 6 |
| 26 | 3 |
| 27 | 3 |
| **Average** | **5.57** |
| **Total** | **117** |

Sleep observations recorded per participant.

Table 3 reports the proportion of missing data for all predictors and covariates included in the analyses. While most variables had minimal missingness (<6%), several behavioral variables presented higher proportions of missing data (~29-35%). Missing observations were excluded from the respective models to ensure valid estimation. Please refer to Table 3 for detailed missing data rates per variable.

**Table 3**

*Missing data per variable*

| **Variable** | **N** | **NA** | **NA (%)** |
| --- | --- | --- | --- |
| Daily sunlight exposure | 113 | 4 | (3.42%) |
| Daily smartphone use | 117 | 0 | (0.00%) |
| Daily step count | 76 | 41 | (35.04%) |
| Before bedtime smartphone use | 117 | 0 | (0.00%) |
| Before bedtime light exposure | 110 | 7 | (5.98%) |
| Before bedtime step count | 117 | 0 | (0.00%) |
| Gender | 117 | 0 | (0.00%) |
| Age group | 117 | 0 | (0.00%) |
| Education level | 110 | 7 | (5.98%) |
| Employment status | 110 | 7 | (5.98%) |
| Household income | 110 | 7 | (5.98%) |
| Children | 110 | 7 | (5.98%) |
| Citizenship | 110 | 7 | (5.98%) |
| BMI category | 117 | 0 | (0.00%) |
| Chronotype | 117 | 0 | (0.00%) |
| Neighborhood | 117 | 0 | (0.00%) |
| Daytime nap | 83 | 34 | (29.06%) |
| Before bedtime food intake | 83 | 34 | (29.06%) |
| Sleep medication | 83 | 34 | (29.06%) |
| Alcohol consumption | 83 | 34 | (29.06%) |
| Caffeine consumption | 83 | 34 | (29.06%) |

*Number of observation and missing data for each variable analyzed in the study*

#### Main Models

**Table 4**

*Associations between sunlight exposure and sleep outcomes*

|  | ***TST*** |  |  | ***N1%*** |  |  | ***N3%*** |  |  |  |
| --- | --- | --- | --- | --- | --- | --- | --- | --- | --- | --- |
| ***Predictors*** | ***Est.*** | ***CI*** | ***p value*** | ***Est.*** | ***CI*** | ***p value*** | ***Est.*** | ***CI*** | ***p value*** |  |
| *(Intercept)* | 410.58 | 335.15, 486.00 | <0.001 | 6.41 | 3.77, 9.05 | <0.001 | 18.14 | 9.39, 26.88 | <0.001 |  |
| *Daily sunlight* | 10.67 | 0.64, 20.70 | 0.037 | -0.29 | -0.55, -0.03 | 0.027 | -0.45 | -1.49, 0.59 | 0.394 |  |
| *Age 30-65* | -53.43 | -121.57, 14.70 | 0.123 | -0.79 | -3.20, 1.61 | 0.516 | 0.10 | -7.84, 8.03 | 0.981 |  |
| *Gender (Male)* | -28.24 | -97.45, 40.96 | 0.420 | 0.81 | -1.64, 3.26 | 0.515 | 0.68 | -7.39, 8.75 | 0.868 |  |
| *Season (vs. autumn)* |  |  |  |  |  |  |  |  |  |  |
| *Spring* | 23.35 | -59.25, 105.94 | 0.420 | -1.05 | -3.86, 1.76 | 0.461 | 1.89 | -7.59, 11.37 | 0.693 |  |
| *Summer* | 62.64 | -9.12, 134.40 | 0.086 | -0.23 | -2.76, 2.31 | 0.859 | 0.93 | -7.43, 9.30 | 0.825 |  |
| *Winter* | 70.37 | -31.98, 172.72 | 0.176 | -0.42 | -4.02, 3.18 | 0.817 | -1.34 | -13.24, 10.56 | 0.824 |  |
| *Weekend (vs. weekday)* | -2.98 | -34.47, 28.52 | 0.852 | 0.05 | -0.73, 0.82 | 0.902 | 1.71 | -1.47, 4.89 | 0.289 |  |
| *Household income (High)* | 7.83 | -50.34, 66.01 | 0.790 | 1.00 | -0.98, 2.98 | 0.320 | 3.17 | -3.51, 9.85 | 0.349 |  |
| *Bed partners (Yes)* | -33.41 | -101.64, 34.81 | 0.334 | 1.47 | -0.89, 3.83 | 0.220 | -0.49 | -8.37, 7.38 | 0.901 |  |
| ***Other information*** |  |  |  |  |  |  |  |  |  |  |
| *σ²* | 4457.95 |  |  | 2.66 |  |  | 45.18 |  |  |  |
| *τ00 ID* | 1743.20 |  |  | 2.72 |  |  | 26.48 |  |  |  |
| *ICC* | 0.28 |  |  | 0.51 |  |  | 0.37 |  |  |  |
| *Marginal R² / Conditional R²* | 0.11 / 0.36 |  |  | 0.11 / 0.56 |  |  | 0.06 / 0.41 |  |  |  |

*Observations: 116; Participants: 21; Est: Estimate of effect size; CI: Confidence intervals (95%); σ²: Residual variance; τ00 ID: Variance between individuals; ICC: Intraclass Correlation Coefficient (variance attributed to individual differences); TST: Total Sleep Time; N1: Stage 1 Sleep; N3: Stage 3 Sleep; Daily sunlight: hours of exposure to light above 1000 lux; Age: 25-29, 30-65; Gender: Female, Male; Season: Spring; Summer, Winter, Autumn; Weekend: Weekend, Weekday; Bed Partner: Yes, No; Household Income (ILS): High (> 5551); Low (≤ 5550).*

**Table 5**

*Associations of artificial light, steps count and smartphone use before bedtime with sleep outcomes*

|  | ***TST*** |  |  | ***SOL*** |  |  | ***N1%*** |  |  | ***N3%*** |  |  |
| --- | --- | --- | --- | --- | --- | --- | --- | --- | --- | --- | --- | --- |
| ***Predictors*** | ***Est.*** | ***CI*** | ***p value*** | ***Est.*** | ***CI*** | ***p value*** | ***Est.*** | ***CI*** | ***p value*** | ***Est.*** | ***CI*** | ***p value*** |
| *(Intercept)* | 348.06 | 235.39, 460.72 | <0.001 | 32.04 | 8.19, 55.88 | 0.009 | 4.54 | 1.46, 7.63 | 0.005 | 20.60 | 8.80, 32.41 | 0.001 |
| *Before bedtime smartphone use* | 0.15 | -0.74, 1.04 | 0.744 | 0.20 | 0.00, 0.39 | 0.047 | 0.01 | -0.01, 0.03 | 0.509 | -0.04 | -0.12, 0.03 | 0.263 |
| *Before bedtime artificial light* | 0.26 | -0.69, 1.21 | 0.588 | 0.02 | -0.19, 0.23 | 0.858 | 0.01 | -0.01, 0.04 | 0.335 | -0.00 | -0.09, 0.08 | 0.982 |
| *Before bedtime step count* | -0.01 | -0.03, 0.02 | 0.559 | 0.00 | -0.00, 0.01 | 0.724 | 0.00 | -0.00, 0.00 | 0.546 | 0.00 | -0.00, 0.00 | 0.080 |
| *Age 30-65* | -49.53 | -139.82, 40.76 | 0.277 | 2.64 | -16.41, 21.70 | 0.782 | -0.21 | -2.70, 2.28 | 0.867 | 0.82 | -8.76, 10.40 | 0.865 |
| *Gender (Male)* | 4.57 | -84.54, 93.67 | 0.919 | -14.97 | -33.74, 3.80 | 0.116 | 1.06 | -1.41, 3.52 | 0.395 | -0.84 | -10.37, 8.69 | 0.861 |
| *Season (vs. autumn)* |  |  |  |  |  |  |  |  |  |  |  |  |
| *Spring* | 137.13 | 26.90, 247.35 | 0.016 | -12.68 | -36.09, 10.73 | 0.283 | -0.68 | -3.67, 2.32 | 0.653 | -0.03 | -11.36, 11.31 | 0.996 |
| *Summer* | 108.31 | 14.69, 201.93 | 0.024 | -15.03 | -34.84, 4.78 | 0.134 | 0.31 | -2.25, 2.88 | 0.808 | -1.28 | -11.10, 8.54 | 0.795 |
| *Winter* | 136.27 | 9.96, 262.57 | 0.035 | 6.68 | -19.99, 33.36 | 0.618 | -0.23 | -3.71, 3.24 | 0.894 | -0.72 | -14.08, 12.64 | 0.915 |
| *Weekend (vs. weekday)* | -19.78 | -67.62, 28.05 | 0.411 | 7.03 | -3.58, 17.65 | 0.190 | 0.45 | -0.73, 1.63 | 0.446 | -0.44 | -4.38, 3.50 | 0.823 |
| *Household income (High)* | -12.35 | -84.25, 59.56 | 0.732 | -0.66 | -15.91, 14.58 | 0.931 | 1.40 | -0.56, 3.36 | 0.158 | 0.75 | -6.71, 8.21 | 0.841 |
| *Bed partners (Yes)* | -17.25 | -104.18, 69.67 | 0.693 | -21.44 | -39.75, -3.13 | 0.023 | 2.35 | -0.05, 4.75 | 0.055 | -3.96 | -13.25, 5.33 | 0.397 |
| ***Other Information*** |  |  |  |  |  |  |  |  |  |  |  |  |
| *σ²* | 5045.09 |  |  | 249.22 |  |  | 3.05 |  |  | 33.68 |  |  |
| *τ00 ID* | 1933.94 |  |  | 79.14 |  |  | 1.69 |  |  | 28.56 |  |  |
| *ICC* | 0.28 |  |  | 0.24 |  |  | 0.36 |  |  | 0.46 |  |  |
| *Marginal R² / Conditional R²* | 0.18 / 0.41 |  |  | 0.36 / 0.50 |  |  | 0.19 / 0.48 |  |  | 0.09 / 0.51 |  |  |

*Observations:* 75*; Participants: 18; Est: Estimate of effect size; CI: Confidence intervals (95%); σ²: Residual variance; τ00 ID: Variance between individuals; ICC: Intraclass Correlation Coefficient (variance attributed to individual differences); TST: Total Sleep Time; N1: Stage 1 Sleep; N3: Stage 3 Sleep; Before bedtime smartphone use: minutes of smartphone use in the 120 minutes before sleep time; Before bedtime artificial light : minutes of exposure to light above 30 lux in the 120 minutes before sleep time. Before bedtime step count: number of steps in the 120 minutes before sleep time; Gender: Female, Male; Season: Spring; Summer, Winter, Autumn; Weekend: Weekend, Weekday; Bed Partner: Yes, No; Household Income (ILS): High (> 5551); Low (≤ 5550).*

To account for potential confounding, we re-estimated the model presented in the main manuscript (Table 6) with additional adjustments for physical activity levels, measured by daily step count (in units of 1,000 steps). Although we believed step count is a critical confounder that has to be adjusted for, we did this in a separate model because, in order to perform this adjustment, we had to further exclude 2 participants and 40 observations overall. In this final model, the initial association between sunlight exposure and TST did not persist. An association between daily step count and TST the following night emerged, with an estimated increase of 6.04 minutes in TST for each 1,000 steps (95% CI: 0.96, 11.12). Comparison of the adjusted and unadjusted models revealed that the point estimate for the relationship between sunlight exposure and TST was not affected, discarding the hypothesis of confounding, but that the width of the 95% CI increased due to the decrease in sample size. The negative association between sunlight exposure and N1 sleep remained, with an estimated reduction of 0.39 percentage points per hour of sunlight exposure (95% CI: -0.72, -0.06). See Table 4 for more details.

**Table 6**

*Associations of daily sunlight exposure and step count with sleep outcomes*

|  | ***TST*** |  |  | ***N1%*** |  |  | ***N3%*** |  |  |
| --- | --- | --- | --- | --- | --- | --- | --- | --- | --- |
| ***Predictors*** | ***Est.*** | ***CI*** | ***p value*** | ***Est.*** | ***CI*** | ***p value*** | ***Est.*** | ***CI*** | **p value** |
| *(Intercept)* | 322.51 | 238.70, 406.32 | <0.001 | 5.43 | 1.45, 9.40 | 0.008 | 20.19 | 9.34, 31.04 | <0.001 |
| *Daily step count* | 6.04 | 0.96, 11.12 | 0.021 | -0.01 | -0.14, 0.12 | 0.907 | -0.21 | -0.69, 0.26 | 0.377 |
| *Daily sunlight* | 11.66 | -0.51, 23.84 | 0.060 | -0.39 | -0.72, -0.06 | 0.021 | 0.03 | -1.14, 1.20 | 0.960 |
| *Age 30-65* | -46.76 | -107.10, 13.59 | 0.127 | -1.41 | -4.49, 1.68 | 0.366 | 1.94 | -6.32, 10.21 | 0.640 |
| *Gender (Male)* | -32.05 | -102.50, 38.40 | 0.367 | 1.28 | -2.07, 4.63 | 0.448 | 1.70 | -7.43, 10.83 | 0.711 |
| *Season (vs. autumn)* |  |  |  |  |  |  |  |  |  |
| *Spring* | 138.63 | 51.21, 226.05 | 0.002 | -0.20 | -4.09, 3.68 | 0.917 | -0.64 | -11.57, 10.30 | 0.908 |
| *Summer* | 123.25 | 53.53, 192.96 | 0.001 | 0.63 | -2.75, 4.00 | 0.712 | -1.98 | -11.13, 7.17 | 0.667 |
| *Winter* | 133.33 | 38.85, 227.80 | 0.006 | 0.67 | -3.88, 5.22 | 0.770 | -1.65 | -14.03, 10.72 | 0.790 |
| *Weekend (vs. weekday)* | -9.99 | -55.96, 35.99 | 0.666 | 0.11 | -1.00, 1.21 | 0.845 | -0.15 | -4.22, 3.92 | 0.942 |
| *Household income (High)* | -20.96 | -77.77, 35.85 | 0.464 | 1.55 | -1.02, 4.11 | 0.233 | 0.87 | -6.29, 8.04 | 0.808 |
| *Bed partners (Yes)* | -40.10 | -107.61, 27.42 | 0.240 | 2.14 | -0.96, 5.23 | 0.172 | -1.76 | -10.30, 6.78 | 0.682 |
| ***Other information*** |  |  |  |  |  |  |  |  |  |
| *σ²* | 4734.36 |  |  | 2.60 |  |  | 35.90 |  |  |
| *τ00 ID* | 687.30 |  |  | 4.02 |  |  | 24.49 |  |  |
| *ICC* | 0.13 |  |  | 0.61 |  |  | 0.41 |  |  |
| *Marginal R² / Conditional R²* | 0.30 / 0.39 |  |  | 0.15 / 0.67 |  |  | 0.06 / 0.44 |  |  |

*Observations: 76; Participants: 19; Est: Estimate of effect size; CI: Confidence intervals (95%); σ²: Residual variance; τ00 ID: Variance between individuals; ICC: Intraclass Correlation Coefficient (variance attributed to individual differences). TST: Total Sleep Time; N1: Stage 1 Sleep; N3: Stage 3 Sleep; Daily step count: number of steps during the day (in units of 1,000 steps); Daily sunlight: hours of exposure to light above 1000 lux; Age: 25-29, 30-65; Gender: Female, Male; Season: Spring; Summer, Winter, Autumn; Weekend: Weekend, Weekday; Bed Partner: Yes, No; Household Income (ILS): High (> 5551); Low (≤ 5550).*

# Bibliography

Choi, L., Liu, Z., Matthews, C. E., & Buchowski, M. S. (2011). Validation of Accelerometer Wear and Nonwear Time Classification Algorithm. *Medicine and Science in Sports and Exercise*, *43*(2), 357–364. https://doi.org/10.1249/MSS.0b013e3181ed61a3

Crouter, S. E., Kuffel, E., Haas, J. D., Frongillo, E. A., & David R. Bassett, J. (2010). A Refined 2-Regression Model for the ActiGraph Accelerometer. *Medicine and Science in Sports and Exercise*, *42*(5), 1029. https://doi.org/10.1249/MSS.0b013e3181c37458

Keadle, S. K., Shiroma, E. J., Freedson, P. S., & Lee, I.-M. (2014). Impact of accelerometer data processing decisions on the sample size, wear time and physical activity level of a large cohort study. *BMC Public Health*, *14*(1), 1210. https://doi.org/10.1186/1471-2458-14-1210
